# Can Care Coordination for Multiple Long‐Term Conditions Be Meaningfully Measured Using Routine Data? A Multi‐Stage Consultation

**DOI:** 10.1111/hex.70894

**Published:** 2026-10-03

**Authors:** Krystal Warmoth, Nicola Small, Vanessa Davey, Patrick Burch, Alex Thompson, Jo Butterworth, Jonathan Gibb, Easter Joury, Steve Callaghan, Felicity Dewhurst

**Affiliations:** ^1^ Academic Centre for Healthy Ageing, Barts Health NHS Trust London UK; ^2^ Queen Mary University of London London UK; ^3^ University of Manchester Manchester UK; ^4^ Newcastle University Newcastle UK; ^5^ University of Exeter Exeter UK; ^6^ Bristol University Bristol UK; ^7^ Patient and Public Contributor, National Institute of Health and Care Research's Multiple Long Term Conditions Cross‐NIHR Collaboration Newcastle UK

**Keywords:** care continuity, care coordination, multimorbidity, quality measurement, quality of care, stakeholder engagement

## Abstract

**Background:**

Care coordination is essential for improving healthcare experiences and outcomes for people living with multiple long‐term conditions (MLTC), yet it remains challenging to measure care coordination in ways that are both meaningful and feasible at scale. Routine electronic health record (EHR) data offer potential for system‐wide measurement, but it is unclear whether such measures reflect what matters to patients, carers, and those delivering care.

**Methods:**

We undertook a multi‐stage, collaborative consultation process to examine whether care coordination in MLTC can be meaningfully measured using routine data. Public contributors (*n* = 18), professional contributors (*n* = 23), researchers (*n* = 15) and data experts (*n* = 10) participated in structured discussions, co‐developed appraisal criteria (including relevance to lived experience and feasibility in EHR data), and assessed measures identified through a linked systematic review. A consensus survey evaluated the appropriateness of shortlisted measures.

**Results:**

Participants identified priorities including teamwork, communication, continuity, care management, and accountability. Appraisal criteria balanced patient relevance with feasibility in routine data. While a feasible shortlist of measures was identified, findings showed only qualified support for routine data–derived indicators. Continuity measures were most strongly endorsed but widely recognised as proxies capturing observable activity rather than the relational, experiential, and cross‐sector dimensions central to coordinated care. Concerns were raised regarding data quality, conceptual validity, and the risk of incomplete or misleading representations.

**Conclusions:**

Routine data–derived measures of care coordination in MLTC hold clear potential but are inherently limited. The findings support a conditional position: such measures can provide useful system‐level signals, but only if interpreted cautiously, supported by robust data infrastructure, and complemented by patient‐reported insights. Advancing measurement will require integrated approaches that better capture what matters to patients and carers.

## 1 Introduction

The number of people with more than one long‐term health condition (MLTC) is increasing globally [1], with significant implications for quality of life and rising demand for health and social care services [2]. The care of individuals with MLTC often involves numerous professionals, agencies, and sectors, making effective care coordination essential for improving outcomes, patient experience, and system efficiency [3, 4, 5, 6, 7, 8, 9]. Despite its recognised importance, people with MLTC frequently experience fragmented care, characterised by poor communication, discontinuity between services, and challenges navigating complex care pathways; these problems are compounded by health inequalities, which can further restrict access to and coordination of care [5, 10, 11].

Given the centrality of coordination to high‐quality care [12], the ability to measure coordination reliably and meaningfully is vital. Numerous care coordination measures exist internationally, but most were not designed with MLTC populations in mind and vary widely in purpose, feasibility, and data requirements [13, 14]. Existing measures of care coordination have limitations, including a tendency to focus on individual services, settings, and observable coordination activities, while under‐representing anticipatory, person‐centred, and self‐management dimensions of care [15]. Many measures were developed for specific conditions or care contexts and may not fully reflect the complexity of coordinating care across multiple providers, organisations, and sectors for people living with MLTC [15]. Evidence suggests that current measures may fail to capture aspects of coordination that are important to contributors, particularly relational and informational dimensions of coordination [13, 16].

Conceptually appealing measures may also be impractical to implement due to documentation burden or limitations within routinely collected electronic healthcare records [17, 18]. Despite sustained investment in national and international initiatives promoting integrated, coordinated, and person‐centred care, including NHS England's Integrated Care Systems [19], NICE multimorbidity guidance [20], the WHO Framework on Integrated People‐Centred Health Services [21], and the UN Decade of Healthy Ageing [22], implementation remains uneven and robust mechanisms for evaluating care coordination in MLTC are still evolving. Importantly, the absence of validated measures limits effective monitoring and evaluation [23, 24]. To be meaningful and workable, decisions about how coordination is assessed must reflect the experiences, priorities, and operational realities of people who use, deliver, and manage services [25].

To address this gap, we undertook a structured collaborative consultation [18] to assess the appropriateness and feasibility of existing measures for MLTC, drawing on a shared definition of care coordination [26].

## 2 Methods

We used a multi‐stage consultation involving discussions, contributor‐informed criteria, and a survey to identify and evaluate measures with contributors (see Figure 1). Informed by the Appropriate measure of Care‐COoRDination for patients with Multiple Long‐Term Conditions (ACCORD) project in the NIHR Cross‐National Collaboration on MLTC Models of Care workstream, we drew on a shared definition of care coordination from Schultz and McDonald [27]:

Care coordination is the deliberate organisation of patient care activities among two or more participants (including the patient) involved in a patient's care to facilitate the appropriate delivery of healthcare services. Organising care involves marshalling the personnel and other resources needed to carry out all required patient care activities, and is often managed through the exchange of information among participants responsible for different aspects of care. [19]

**Figure 1:**
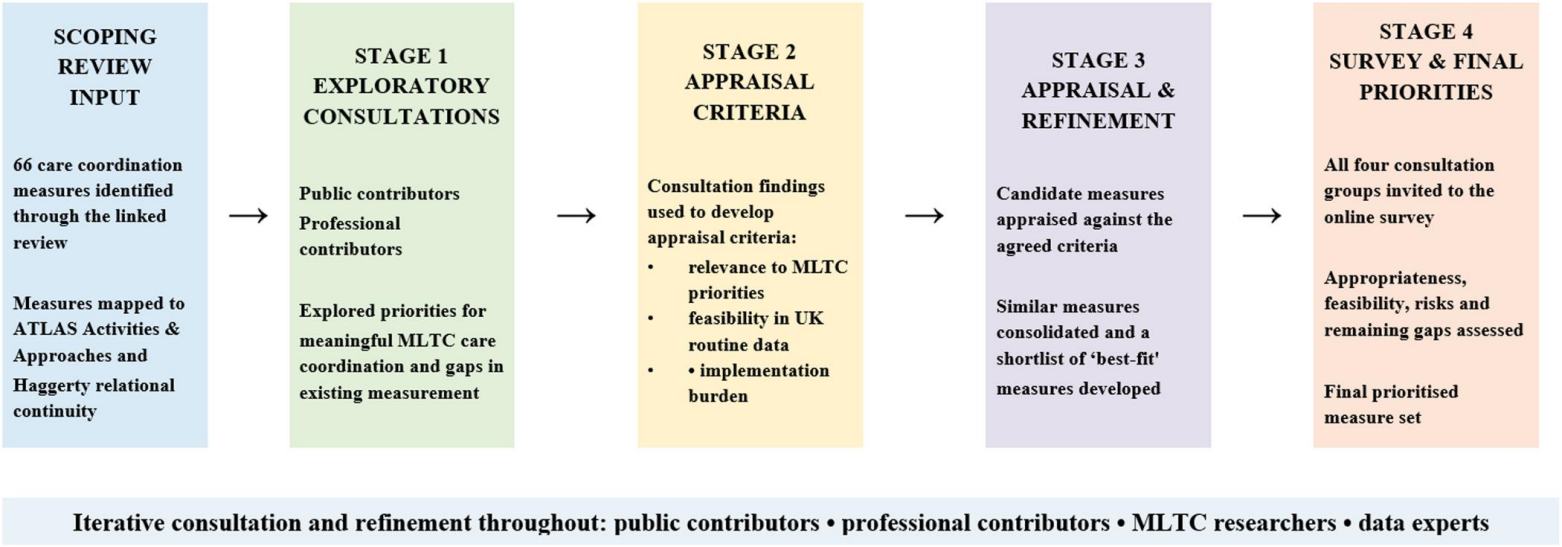
Multi‐stage consultation and measure appraisal process.

### 2.1 Participants and Contributors

We consulted four groups with complementary expertise through separate consultation activities: public contributors with lived experience of MLTC; professional contributors involved in designing, delivering, or commissioning care; MLTC researchers; and data experts specialising in electronic health records (EHRs) and routinely collected data. All participated in structured consultations using comparable materials. Table 1 reports the number of unique participants across contribution groups.

**Table 1:** Participant and contributors’ characteristics across the different consultation groups.

Public contributors, *n* = 18		Professional contributors, *n* = 23		Researchers, *n *= 15	
*Gender*					
Male	6	Male	8	Male	6
Female	12	Female	15	Female	9
*Geographical location*					
London	3	London	10	London	0
West Midlands	4	West Midlands	3	West Midlands	2
East Midlands	2	East Midlands	1	East Midlands	0
East of England	3	East of England	2	East of England	1
North West England	2	North West England	2	North West England	3
North East England	2	North East England	1	North East England	1
South central England	0	South central England	0	South central England	2
South East England	0	South East England	0	South East England	1
Southwest England	1	Southwest England	4	Southwest England	3
Scotland	1	Scotland	0	Scotland	2
*Ethnicity*		*Main role/sector*		*Professional background*	
White British	10	Health or social care practitioner	4	Geriatrics	2
Ethnic minority group	8	Policy or commissioning representative	6	General Practice	2
		Charity of voluntary sector representative	6	Psychiatry	1
		Health and care consultancy	2	Palliative Care	1
		Methods expert	1	Physiotherapy	1
		Research funder representative	1	Nursing	1
		Public representative	3	Pharmacy	1
				Psychology	1
				Health services research	1
				Health economics	1
				Epidemiology	1
				Statistics	1
				Patient and public involvement	1

#### 2.1.1 Public Contributors

Eighteen public contributors were recruited from an established MLTC Patient and Public Involvement and Engagement (PPIE) panel. They had lived experience of managing MLTC, caring for someone with MLTC, or supporting people with complex needs. They also had previous experience with public and patient involvement. The contributors took part in three structured discussion sessions to explore what good care coordination should look like, which aspects of coordination were most important to them, and how existing measurement approaches aligned with or conflicted with their experiences.

#### 2.1.2 Professional Contributors

A stakeholder mapping exercise was undertaken to identify, categorise, and prioritise individuals and groups with a substantive interest in the development, delivery, implementation, or outcomes of models of care for people living with MLTC [28]. Stakeholders were assessed according to their levels of influence (defined as the ability to shape decisions, allocate resources, or affect implementation at scale) and interest (reflecting the degree to which stakeholders are directly affected by, involved in, or have a vested interest in models of care). Using these criteria, contributors were situated within a four‐quadrant influence‐interest matrix, corresponding to different levels of engagement (actively engage, keep satisfied, keep informed, and monitor). To support engagement planning, the matrix was operationalised as a structured stakeholder mapping tool that captured stakeholder role (e.g. influencer, regulator, or implementer), area of influence or interest, relevance to different phases of the work, engagement lead, engagement approach, engagement tools, and frequency of engagement. Recruitment for the priority‐setting consultation was informed by this mapping and prioritised contributors identified for active engagement, particularly those with high relevance to MLTC care coordination measurement and implementation and higher levels of influence and/or interest, while also seeking representation across a range of professional and organisational perspectives. The mapping outcomes additionally informed wider engagement activities, including participation, survey dissemination, communication strategies, and dissemination planning, with stakeholders in lower influence‐interest categories identified for ongoing monitoring or targeted sharing of study findings. The stakeholder map was reviewed iteratively throughout the project to support engagement, recruitment, communication, and dissemination activities.

Two online consultation sessions were held for professional contributors to attend, who participated both as representatives of their organisations and in their individual capacities. The first session was attended by 21 people, and 13 people attended the second session. Of the people attending the second session, 2 had not attended the first session. We engaged 23 professional contributors, including policy or commissioning representatives (*n* = 6), charity or voluntary‐sector representatives (*n* = 6), health or social care practitioners (*n* = 4), public representatives (*n* = 3), health and care consultants (*n* = 2), a methods expert (*n* = 1), and a research funder representative (*n* = 1) (Table 1). Like the public contributors, they participated in structured discussions and completed similar sets of tasks, focusing on priorities for measuring coordination, perceived gaps in current approaches, and the practical implications of different types of measures within real‐world service constraints.

#### 2.1.3 MLTC Researchers

Members of the wider MLTC Models of Care workstream, including 15 researchers, also participated in the consultation process. Team members brought expertise in practice and policy, and current research priorities in MLTC. They engaged with the same materials and tasks as the public and professional contributor groups, reviewed the developing appraisal criteria, and contributed to the refinement of the consultation tools and best‐fit measure shortlist. Their role was both analytic and integrative, helping to ensure that experiential, professional and system‐level perspectives were coherently represented throughout the consultation process.

#### 2.1.4 Data Experts

Ten data experts had professional experience in EHRs, health informatics, data linkage, coding practices, and routinely collected datasets. They represented a range of settings, including academia, NHS and integrated care, local government, health data infrastructure, and health technology and analytics organisations, and included both methodological and clinical expertise (Table 1). They contributed insights into feasibility, data availability, documentation practices, and issues associated with operationalising diverse measures within UK routine data systems.

### 2.2 Stage 1: Exploratory Consultations

We conducted a series of interactive online consultation sessions with public and professional contributors to explore how care coordination is experienced and understood within MLTC. The first consultation examined (a) understandings of care coordination, (b) its importance and barriers to care in an MLTC context, and (c) prioritisation of domains within a well‐established framework (the Agency for Healthcare Research and Quality Care Coordination Measurement Atlas or ATLAS) [29]. During the second consultation, the value of electronic health and care records and the differences between public and professional contributors’ perspectives were discussed. The third consultation considered the best‐fit measures for the consensus survey (Stage 4).

Notes from all sessions were collated. Responses were collected from the videoconferencing transcripts, chat platform, and an audience engagement tool, drawing on participant contributions and discussions shared throughout the sessions.

### 2.3 Stage 2: Development of Appraisal Criteria

Drawing on the insights generated during Stage 1, we developed a set of appraisal criteria to assess candidate measures identified through a linked scoping review [15] and evaluated how well each measure reflected the priorities articulated during exploratory consultations. This included assessing conceptual fit with the issues that public and professional contributors identified as important to measure. Together, these criteria were selected to ensure that selected measures balanced meaningfulness, burden, and operational feasibility.

### 2.4 Stage 3: Appraisal of Candidate Measures and Refinement

A list of 64 measures identified through the scoping review [15] was first mapped to the ATLAS Activities and Approaches [29], and to Haggerty's relational continuity domain [16] to ensure comprehensive conceptual coverage. An additonal three measures were not identified through the scoping review: referral loop completeness [36], medication reconciliation within 14 days of discharge or transition [55], and the polypharmacy review rate [56]. These were proposed by the review team on the basis of UK electronic record practice and discussion with the data experts. Each mapped measure was then appraised using the criteria developed in Stage 2. This appraisal resulted in an initial set of “best‐fit” measures for each domain, defined as those measures that most strongly aligned with MLTC priorities while remaining feasible for implementation in UK data systems. Measures capturing similar constructs were consolidated to improve clarity, reduce duplication, and enhance interpretability.

### 2.5 Stage 4: Survey of All Consultation Groups

To further refine and assess the revised set of best‐fit measures, we developed an online anonymous survey to gather additional perspectives from those involved in earlier stages (see Supporting Information). All four consultation groups were invited to participate via the survey mailing list (*n* = 72), comprising public contributors (*n* = 16), professional contributors (*n* = 20), MLTC researchers (*n* = 24), and data experts (*n* = 12). The survey mailing list comprised 72 individuals and was broader than attendance at the preceding consultation activities; therefore, this number does not represent the sum of participants reported for the earlier consultation stages. The survey tested the robustness of the appraisal process by validating the criteria‐based appraisal conducted in the previous stage and identifying any remaining uncertainties or gaps requiring further deliberation.

Participants were asked to evaluate each shortlisted measure across several dimensions: the overall appropriateness of each measure for capturing care coordination in MLTC; comment on the feasibility and acceptability of implementing the measures in practice; and to identify any risks associated with their use. The survey sought input on whether any important aspects of care coordination remained insufficiently captured. Participants were encouraged to highlight gaps in current measurement approaches and to suggest additional domains or constructs that may warrant attention in subsequent stages of development. Participants were also asked whether they had one or more long‐term health conditions (Yes/No/Prefer not to say). This question was included to identify participants with lived experience of long‐term conditions; it did not distinguish between individuals living with a single long‐term condition and those living with multiple long‐term conditions.

Due to a technical issue in Qualtrics, 22 invitees received a version of the survey in which comparison‐rating items were omitted. These individuals were subsequently sent a follow‐up questionnaire containing only the missing items. Responses to the corrected survey were analysed alongside the main survey responses to assess consistency. Questions included in the follow‐up survey are presented in the Supplementary Materials. Quantitative data were analysed using IBM SPSS Statistics, version 29. The analysis was descriptive, displaying the proportions of agreement with the appropriateness of the measure and the output of the priority setting exercises. Percentages were calculated using the number of valid responses to each survey item as the denominator, unless otherwise specified. Only completed questionnaires were included in the final analysis. Core survey items were set as required, such that participants were required to provide a response before progressing to the next question; demographic questions and free‐text comments were optional. Missing responses to optional items were excluded from item‐level analyses, and descriptive statistics were calculated using available data for each variable. No weighting or statistical adjustment was applied to the descriptive survey results. Mean ranks were used as a descriptive summary to facilitate comparison of relative priorities across items and are presented for descriptive purposes only, recognising the ordinal nature of the ranking data. The free‐text comments were extracted and managed using Excel. Across the main survey, 403 free‐text comments were provided, with a further 21 comments provided in the follow‐up survey, in which free‐text responses were optional. Comments were read and manually coded, using an inductive approach informed by reflexive thematic analysis [30]. Initial descriptive codes and themes were generated from individual comments, with related themes iteratively reviewed and grouped into broader analytical themes through discussion among members of the research team. The final analytical themes were refined collaboratively and used to structure the presentation of the free‐text findings.

### 2.6 Ethics and Involvement Principles

The work followed institutional involvement guidance and adhered to the UK Standards for Public Involvement [31], particularly related to working together, inclusive opportunities and governance (see Supplementary materials). Participants were given thank you payments in recognition of time, skills, and expertise. Although the project concerned consultation and prioritisation rather than collection of personal or clinical data, Newcastle University Ethics Committee approval (NU‐01414) and informed consent were obtained for the survey. Quotes were used for illustrative purposes.

## 3 Results

### 3.1 Stage 1: Exploratory Consultations

#### 3.1.1 Conceptualisation and Priorities for Care Coordination and Continuity

Participants shared a broadly consistent view of care coordination for people living with MLTC, describing it as a joined‐up, person‐centred process spanning conditions, services, and organisational boundaries. Coordination was seen as essential to reflecting patient and carer experiences, with effective communication, clear pathways, and integrated information systems critical to reducing fragmentation and duplication. The inherent complexity of MLTC—including polypharmacy and evolving needs—was emphasised, alongside the need for measures that are actionable, accountable, and sensitive to change over time.

Priority domains identified through ranking exercises included teamwork, communication, care management, monitoring and follow‐up, and assessment of needs and goals. These align with a view of coordination as dependent on multidisciplinary collaboration and seamless information flow across health and social care.

Continuity of care, although explicitly an ATLAS domain, emerged as a core component. Participants highlighted the importance of consistent professionals or stable teams in building trust, avoiding repeated assessments, and supporting coherent long‐term management. Consequently, relational continuity [16] was incorporated, defined as an ongoing therapeutic relationship between patients and care providers over time.

EHRs were recognised as valuable for capturing process‐based indicators (e.g. contacts, care planning, transitions, accountability), offering structured and routinely collected data across settings. However, their limitations were clear: they do not capture patient experience, perceived quality, or wider impacts on wellbeing. Participants therefore emphasised the need to complement EHR data with patient‐reported outcome measures (PROMs) or patient‐reported experience measures (PREMs). Crucially, measures should be validated across patient, carer, and provider perspectives to avoid superficial, system‐centred outputs and ensure they reflect meaningful aspects of coordinated care.

#### 3.1.2 Distinct Priorities in Defining and Measuring Coordination

Despite broad consensus across contribution groups, important differences in emphasis were evident. Patients and carers prioritised trust, relational continuity, carer involvement, and visibility of services, while highlighting the risks of burden‐shifting—where coordination responsibilities are placed on patients—and raising concerns about digital exclusion and inequities in access to and benefit from care coordination.

Professional contributors focused more strongly on accountability at meso‐ and macro‐levels, spanning organisations, systems, and networks. They emphasised the role of prevention and the value of measurement in identifying individuals in need of support and detecting early signs of poor coordination. At the same time, they highlighted tensions between cost and quality, and ongoing challenges in resource allocation.

MLTC researchers placed particular emphasis on methodological rigour, favouring measures with established validity, reliability, and comparability. They stressed the importance of balancing process indicators with outcome measures to capture both how coordination occurs and its impacts. They underlined the need for definitional clarity to avoid conceptual ambiguity and to enable meaningful benchmarking across services and systems.

Taken together, these perspectives underscore the need for coordination measures that are both methodologically robust and responsive to the lived experiences of those navigating coordinated care.

### 3.2 Stage 2 and 3: Appraisal of Candidate Measures Against Contributor‐Informed Criteria

#### 3.2.1 Development of Appraisal Criteria

The appraisal framework was grounded in shared priorities and distinct emphases expressed across contribution groups (Table 2). Criteria assessed (1) relevance to MLTC priorities, (2) feasibility in UK routine data, and (3) implementation burden, balancing meaningfulness with practicality. Across the consultations, patients, carers, clinicians, and researchers consistently valued measures that captured coordination across multiple conditions and settings, reflected patient and carer experience, and minimised additional burden. Together, these criteria enabled systematic identification of best‐fit measures aligned with contributor priorities, feasible within UK data systems, and minimising burden. The framework provided a structured basis for selecting conceptually robust and practically implementable measures of MLTC care coordination.

**Table 2:** Appraisal framework for care coordination measures in MLTC.

Domain	Criterion	Description	Assessment Indicators
Contributor priorities	Scope across conditions	Captures coordination across multiple conditions	Reflects MLTC; reduces fragmentation
	Cross‐setting coordination	Integration across care settings	Captures transitions and communication
	Patient‐level focus	Represents patient perspective	Reflects whole care journey
	Complexity and polypharmacy	Accounts for multiple providers/medications	Identifies duplication and burden
	Patient and carer relevance	Relational and experiential care	Trust, support, involvement
	Equity and inclusivity	Addresses inequalities	Captures variation across groups
	Accountability	Clarity of responsibility	Identifies coordination ownership
	Actionability	Supports improvement	Drives intervention
	Dynamic needs	Captures care over time	Reflects transitions and change
Feasibility in UK data	Data availability	Presence in routine datasets	High/Medium/Low
	Data quality	Consistency of coding	Standardised vs inconsistent
	Granularity	Timing and sequencing	Precise vs. absent
	Linkage	Ability to connect datasets	High vs. siloed
Minimising burden	Burden of adaptation	Effort to implement measure	Low/Moderate/High

#### 3.2.2 Refinement and Consolidation of Best‐Fit Measures for Prioritisation

The initial appraisal identified multiple best‐fit measures across ATLAS domains, many capturing similar constructs but differing in operational detail or domain mapping. Discussions with the research and MLTC CNC teams established the need for consolidation to reduce duplication, avoid over‐weighting constructs, and improve interpretability. Measures presented to data experts aligned with the final measure families, and subsequent changes reflected planned consolidation rather than omissions.

Refinement was guided by principles of conceptual clarity, non‐duplication, coverage of core coordination functions, preference for direct measures, feasibility within UK electronic health records, interpretability, resource requirements, and recognition of gaps. Reassessment of conceptual fit ensured measures were retained only where alignment with domain definitions was strong.

Refinement prioritised direct, feasible, and interpretable indicators across continuity, transitions, referrals, medication management, teamwork, and complexity, with relational continuity treated as a distinct construct. Table 3 presents the initial and final measure sets and the rationale for selection. Measures not retained were considered valid but excluded to maintain parsimony and balance.

**Table 3:** Initial best‐fit measures for care coordination domains (pre‐prioritisation).

Domain	Best‐Fit Measure	Reason for Selection
Accountability: Establish accountability/negotiate responsibility	Known Provider Continuity—personal provider (KPC‐PP) [32]	Sustained personal provider relationship across years; reflects ongoing responsibility; moderate UK feasibility
	Usual Provider of Care (UPC) [33]	Simple and interpretable proxy for responsibility and continuity; highly feasible in UK data
Accountability: Align resources with patient and population needs	Specialty Intensity Score (SIS) [34]	Estimates specialist needs; supports stratification; highly compatible with UK data.
Communication: Interpersonal communication	Continuity of GP care (CGPC/GP‐restricted continuity index) [33]	Captures continuity in GP care; strong proxy for relationship‐based communication.
	Sequential Encounter Continuity (SECON) [35]	Captures specialist continuity; reflects relational communication in secondary care.
	Known Provider Continuity ‐ Specialist Provider (KPC‐SP) [32]	Captures persistent specialist relationships over time; feasibility depends on identifiers.
Communication: Information transfer	Referral loop completeness (closed‐loop referral) [36]	Measures closed‐loop referrals; directly captures communication quality.
	Discharge communication (timely discharge summary/transition record) [37]	Measures timely transfer of discharge summaries; key for transitions.
Transitions: Across settings	Follow‐Up After Hospitalisation (FUH‐7/30) [38]	Measures follow‐up after discharge; widely used and actionable.
Transitions of care: As coordination needs change	Cover Index [39]	Captures gaps in care over time; reflects evolving needs.
Care planning: Assess needs and goals	Specialty Intensity Score (SIS) [34]	Proxy for complexity and care planning; feasible at scale.
Care planning: Monitor, follow up, and respond to change	Abnormal results follow‐up [40]	Measures timely response to abnormal results; clinically meaningful and safety focused.
Teamwork ‐ Interdisciplinary collaboration	Care Density [41]	Measures provider interconnectedness as a proxy for teamwork; complements continuity in MLTC care.
Care Management	Fortney Continuity (Case Management Continuity) [42]	Directly captures sustained case‐management involvement, the core of care management; aligns with MLTC needs, with moderate UK feasibility.
	Coordination risk score [43]	Captures cross‐condition coordination (e.g., follow‐up, medication oversight, communication), reflecting active multimorbidity management; UK feasibility is moderate–high.
Medication Management	Medication reconciliation within 14 days of discharge/transition [55]	Measures timely medication reconciliation after transitions; high value for MLTC safety, with moderate UK feasibility depending on structured coding.
	Polypharmacy review rate [56]	Measures annual review in polypharmacy patients; captures optimisation and deprescribing in high‐risk MLTC populations, complementing reconciliation.
Health IT‐enabled coordination	Electronic Health Information Exchange/Electronic Medical Record use [37]	Captures active information exchange supporting coordination and continuity; UK feasibility is moderate, with discharge communication as a proxy.
Health Care Home	Medical group continuity (attribution stability) [44]	Provides a stable organisational “home” aligned with MLTC care; more feasible in UK data, and captures organisational responsibility beyond clinician‐level continuity.
Relational Continuity	Known Provider Continuity—personal provider (KPC‐PP) [32]	Captures sustained patient–provider relationships over time, aligning with relational continuity and reflecting ongoing care responsibility.

### 3.3 Stage 4: Survey of ‘Best‐Fit’ Measures With All Consultation Groups

#### 3.3.1 Participant Characteristics

The survey was distributed to a mailing list of 72 individuals across the four consultation groups: public contributors, professional contributors, MLTC researchers, and data experts. Of these, 37/72 responded (51% response rate). Owing to a technical issue in Qualtrics, 22 invitees received a version of the survey that omitted the comparison‐rating questions. These participants were subsequently sent a follow‐up questionnaire containing only the missing items, of whom 15/22 responded (68.0% response rate). Participants were predominantly aged 35–54 years (*n* = 18/32, 56.3%). Gender was evenly distributed, with 15/32 (46.9%) identifying as female and 15/32 (46.9%) as male, while 2/32 (6.3%) did not disclose their gender. Regarding health status, 13/32 (40.6%) of respondents reported living with a long‐term condition, whereas 15/32 (46.9%) did not. Most participants identified as White British (19/32, 57.6.%), with smaller representation across other ethnic groups or choosing not to disclose their ethnicity. Table 4 summarises the integration of survey findings and consultation insights across key domains of care coordination and the corresponding best‐fit measures.

**Table 4:** Integration of contributor perspectives and survey findings across care coordination domains and best‐fit measures.

Domain	Best‐Fit Measure	Perceived Appropriateness	Contributor Insights	Interpretation
Accountability—Establish Accountability or Negotiate Responsibility	Known Provider Continuity—personal provider (KPC‐PP) [32]	59.5% yes	Emphasis on clarity of responsibility; concerns about capturing true coordination vs. continuity	Strong support, but the measure may reflect continuity rather than broader accountability
		21.6% no		
		18.9% Unsure		
Accountability—Align resources with patient and population needs	Usual Provider of Care (UPC) [33]	16.7% very appropriate	Recognition that service intensity may reflect complexity and need; concerns that higher utilisation may indicate fragmented rather than coordinated care.	Moderate support, but the measure may capture healthcare utilisation more directly than resource alignment or coordination.
		66.7% somewhat appropriate		
		16.7% neither appropriate nor inappropriate		
	Specialty Intensity Score (SIS) [34]	1/6 (16.7%)^a^ very appropriate	Viewed as reflecting consistency of provider involvement, uncertainty about its relevance to patient experience and accountability.	Moderate support, but the link between provider coverage and meaningful coordination remains unclear.
		4/6 (66.7%) somewhat appropriate		
		1/6 (16.7%) neither appropriate nor inappropriate		
Communication ‐Interpersonal Communication	Continuity of GP care (CGPC/GP‐restricted continuity index) [33]	3/7 (42.9%) very appropriate	Ongoing relationships were associated with trust and better communication; concerns that continuity does not directly measure communication quality.	Strong support, but the measure reflects opportunities for communication rather than communication itself.
		3/7 (42.9%) somewhat appropriate		
		1/7 (14.3%) not appropriate		
	Sequential Encounter Continuity (SECON) [35]	3/7 (42.9%) very appropriate	Familiarity and trust were valued; concerns that continuity alone does not demonstrate effective communication.	Generally supported, but the measure captures continuity more directly than interpersonal communication.
		2/7 (28.6%) somewhat appropriate		
		1/7 (14.3%) neither appropriate nor inappropriate		
		1/7 (14.3%) not appropriate		
	Known Provider Continuity—Specialist Provider (KPC‐SP) [32]	2/7 (28.6%) very appropriate	Concerns that continuity with a single specialist may be less relevant for people requiring input from multiple specialists.	Mixed support, suggesting the measure may inadequately capture communication across multidisciplinary teams.
		1/7 (14.3%) somewhat appropriate		
		4/7 (57.1%) somewhat inappropriate		
Communication—Information Transfer	Referral loop completeness (closed‐loop referral) [36]	3/7 (42.9%) very appropriate	Importance of information flowing between services; concerns that completed referrals do not demonstrate effective communication.	Generally supported, but captures administrative completion rather than the quality of information transfer
		2/7 (28.6%) somewhat appropriate		
		2/7 (28.6%) neither appropriate nor inappropriate		
	Discharge communication (timely discharge summary/transition record) [37]	2/7 (28.6%) very appropriate	Viewed as important for safe transitions, concerns about variation in quality, completeness, and follow‐through.	Moderate support, but documentation of communication may not reflect whether information was effectively used.
		2/7 (28.6%) somewhat appropriate		
		1/7 (14.3%) neither appropriate nor inappropriate		
		1/7 (14.3%) somewhat inappropriate		
		1/7 (14.3%) not appropriate		
Transitions of care: Across settings	Follow‐Up After Hospitalisation (FUH‐7/30) [38]	70.3% yes	Strong emphasis on preventing gaps in care following discharge; follow‐up viewed as important for continuity and safety.	Strong support, although follow‐up alone does not demonstrate the quality or effectiveness of transitional care.
		10.8% no		
		18.9% unsure		
Transitions of care: As coordination needs change	Cover Index [39]	35.1% yes	Recognition of the need for measures that reflect changing care needs; uncertainty about whether provider coverage captures adaptation over time.	Considerable uncertainty suggests the measure may not adequately reflect responsiveness to changing coordination needs.
		27.0% no		
		37.8% unsure		
Care planning: Assess needs and goals	Specialty Intensity Score (SIS) [34]	51.4% yes	Complex needs were recognised as requiring greater service involvement; concerns that intensity does not reflect patient goals or preferences.	Moderate support, but the measure captures complexity more directly than person‐centred care planning.
		24.3% no		
		24.3% unsure		
Care planning: Monitor, follow up, and respond to change	Abnormal results follow‐up [40]	59.5% yes	Viewed as an important indicator of responsiveness and patient safety, concerns regarding variation in clinical relevance.	Strong support, although the measure captures a specific aspect of follow‐up rather than broader care planning processes.
		13.5% no		
		27.0% unsure		
Patient Centredness ‐ Support Self‐Management Goals	None found	—	Strong emphasis on understanding patient priorities, supporting self‐management, and ensuring patients feel listened to and involved in care decisions. Participants considered these aspects central to coordinated care.	No suitable routine EHR‐based measure was identified, highlighting a significant gap between what contributors value and what current measures capture.
Patient Centredness—Link to Community Resources	None found	—	Participants emphasised the importance of social prescribing, community services, voluntary sector support, carer involvement, and addressing wider determinants of health.	No suitable measure was identified, suggesting existing coordination measures inadequately capture community‐based and holistic support.
Teamwork—Interdisciplinary collaboration	Care Density [41]	54.1% yes	Shared involvement of providers was seen as a potential marker of collaboration; concerns that shared patients do not necessarily indicate teamwork.	Moderate support, but the measure may overestimate collaboration between professionals.
		18.9% no		
		27.0% unsure		
Care Management	Fortney Continuity (Case Management Continuity) [42]	2/7 (28.6%) very appropriate	Strong emphasis on having a coordinating role and clear responsibility for managing complex care.	Strong support, reflecting contributor priorities around accountability, navigation support, and continuity.
		4/7 (57.1%) somewhat appropriate		
		1/7 (14.3%) somewhat inappropriate		
	Coordination risk score [43]	2/7 (28.6%) very appropriate	Relevant to the challenges of MLTC but concerns that disease‐focused indicators may not reflect coordination experiences.	Mixed support, suggesting the measure may capture clinical management more effectively than care coordination.
		2/7 (28.6%) somewhat appropriate		
		3/7 (42.9%) somewhat inappropriate		
Medication Management	Medication reconciliation within 14 days of discharge/transition [55]	1/7 (14.3%) very appropriate	Viewed as important for reducing medication errors and supporting safe transitions.	Generally supported, but completion of reconciliation does not necessarily indicate quality or effectiveness.
		4/7 (57.1%) somewhat appropriate		
		1/7 (14.3%) neither appropriate nor inappropriate		
		1/7 (14.3%) somewhat inappropriate		
	Polypharmacy review rate [56]	2/7 (28.6%) very appropriate	Medication review was considered highly relevant for people with MLTC; concerns focused on variation in review quality.	Moderate support, although the measure reflects review activity rather than patient benefit.
		3/7 (42.9%) somewhat appropriate		
		2/7 (28.6%) somewhat inappropriate		
Health IT‐enabled coordination	Electronic Health Information Exchange/Electronic Medical Record use [37]	62.2% yes	Strong emphasis on information sharing and interoperability; concerns about fragmented systems and incomplete data exchange.	Strong support, but technology use alone does not demonstrate effective coordination.
		13.5% no		
		24.3% unsure		
Health Care Home	Medical group continuity (attribution stability) [44]	51.4% yes	Stable organisational responsibility was viewed as potentially beneficial; uncertainty remained about its relevance to patient experience.	Moderate support, but organisational continuity may not translate into perceived coordination.
		29.7% no		
		18.9% unsure		
Relational Continuity	Known Provider Continuity—personal provider (KPC‐PP) [32]	73.0% yes	Importance of trust, familiarity, and not having to repeat information; continuity viewed as central to coordinated care.	Strong support, reflecting contributor priorities around relationship‐based care, although the measure captures continuity more directly than coordination.
		10.8% no		
		16.2% unsure		

*Note:* Measures are ordered broadly according to the level of support reflected in the survey findings and consultation insights, from stronger support to greater uncertainty or identified measurement gaps; Shading indicates the overall interpretation of support: green = strong support; amber = moderate or general support; red = mixed or uncertain support; grey = identified measurement gap.

^a^ Frequencies and valid‐response denominators are reported alongside percentages for measures based on small subgroup counts (*n* = 6–7).

#### 3.3.2 Variation in Support for Measures

Responses showed clear variation in support across coordination measures, with the strongest endorsement for those capturing continuity and relationship‐based care. GP Continuity and Care Management Continuity were each rated appropriate by most of the respondents, while the Specialty Intensity Score and Relational Continuity were also highly supported. These results indicate a clear preference for measures reflecting sustained interactions and ongoing relationships.

System‐level process measures received more mixed support. Referral loop completion (closed loop referrals) and medication reconciliation were each endorsed by over 70% of respondents, and the cover index by about two‐thirds, while discharge communication received lower support. Although generally acceptable, these measures were viewed as less consistently fit‐for‐purpose than continuity‐focused measures. Some measures attracted more disagreement. Specialist continuity was rated inappropriate by more than half of respondents, and comorbidity indicators were rated inappropriate by over 40%, suggesting misalignment with lived experiences of coordination. Measures of coordination across care settings were relatively well supported, while responsiveness to changing needs showed considerable uncertainty. Broader domains such as accountability and monitoring, teamwork, and healthcare home received moderate support.

When ranking domains by importance (with lower mean ranks indicating higher priority), respondents placed greatest emphasis on care management (mean rank = 2.17), followed by teamwork (2.46), medication (2.74), health IT (3.23), and accountability (3.78), reflecting a focus on core clinical coordination (i.e., coordination processes within and between healthcare professionals and services) and system infrastructure. Mid‐ranked domains included information transfer, transitions, and proactive planning, suggesting their role as supporting functions. Self‐management (7.57) and community resources (9.62) were ranked lowest, indicating comparatively lower priority. Overall, these findings suggest a preference for strengthening internal, clinically focused coordination processes, with comparatively less emphasis on coordination activities led by patients or delivered through community‐based resources.

#### 3.3.3 Addressing Proxy, Data, and Validity Challenges

The free‐text responses extended and deepened the quantitative findings regarding the appropriateness and support for proposed measures. While respondents broadly endorsed the principle of measuring care coordination using EHR‐based indicators, they consistently raised concerns about what these measures capture, how they are constructed, and whether they meaningfully reflect the underlying construct of care coordination.

Three interrelated challenges emerged: reliance on proxy measures, structural limitations in the data systems underpinning these measures, and more fundamental issues concerning conceptual validity. Together, these themes highlight a tension between the practical feasibility of measurement and the complexity of care coordination, particularly for people with MLTCs. While some indicators were viewed as useful, pragmatic approximations, others were considered insufficiently sensitive—or misleading—in capturing the multidimensional nature of coordinated care. These findings were synthesised into three overarching analytical themes, presented below.

##### 3.3.3.1 Measures Are Useful But Incomplete Proxies for Complex, Person‐Centred Coordination

Respondents consistently characterised many of the proposed indicators as pragmatic proxy measures—operationally feasible, process‐based metrics that indirectly capture aspects of care coordination. Their value lay in their routine availability within EHR systems and their scalability for system‐level monitoring. However, these measures were widely understood to capture observable activity rather than the quality, effectiveness, or experience of coordination.

Continuity‐based indicators (e.g., Usual Provider of Care, Known Provider Continuity, Continuity of GP Care) were frequently cited as examples of such proxies. These were often regarded as meaningful and practical starting points, particularly because they are well‐established and relatively easy to operationalise. However, respondents consistently questioned whether continuity alone reflects critical dimensions of coordination, such as communication, shared accountability, and collaborative working. As one participant noted:

I don't see how continuity of care reflects interpersonal communication.

(R8)

Similarly:

Continuity does not show communication quality.

(R19)

These accounts suggest that, while continuity indicators offer a useful approximation, they systematically under‐represent the relational, interpersonal, and organisational components of coordination. This limitation becomes particularly pronounced for people with MLTCs, whose care typically involves multiple professionals and services across organisational boundaries. In such contexts, continuity with a single provider risk oversimplifying the realities of coordinated care.

More broadly, responses indicate that current measurement approaches are shaped by what is readily quantifiable, resulting in partial and reductionist representations of coordination. As one participant succinctly observed:

‘Done’ doesn't mean ‘done well’.

(R26)

Respondents highlighted that existing indicators are disproportionately centred on healthcare system activity, with limited visibility of coordination across social care, community services, and wider determinants of health. As a result, these measures may fail to capture coordination that occurs across organisational and sectoral boundaries.

Despite these limitations, proxy indicators were not dismissed outright. Instead, respondents positioned them as useful but incomplete tools—capable of providing high‐level signals of coordination when interpreted cautiously and supplemented with other sources of information. However, concerns extended beyond what is measured to the reliability of the data used to construct these indicators.

##### 3.3.3.2 Routine Data and System Constraints Limit Reliable Measurement of Coordination

Respondents raised substantial concerns about the quality and reliability of the underlying data used to derive coordination measures. These concerns included inconsistencies in recording practices, missing or incomplete data, and variability across organisations and systems. Such issues were seen as directly undermining the robustness of any resulting indicators. As one participant reflected:

I don't know if I trust healthcare data to be accurately recorded.

(R36)

Taken together, these issues suggest that even well‐designed indicators may be compromised by weaknesses in the underlying data infrastructure.

A central and recurring concern was the lack of interoperability between systems spanning healthcare, social care, and other sectors. Participants repeatedly identified fragmented information systems as a critical limitation:

Information flow of this type is not routine.

(R13)

Different computer systems are unable to talk to each other.

(R10)

These accounts highlight that fragmented data systems not only hinder effective coordination in practice but also constrain its measurement. Without integrated records and seamless data sharing, coordination cannot be comprehensively observed or assessed.

Respondents further pointed to structural and organisational constraints—including misaligned incentives, resource limitations, and variation in local data infrastructures—as barriers to developing meaningful metrics. Improving measurement was therefore seen as contingent on strengthening data systems, including the development of linked datasets across sectors, standardised coding practices, and more robust information‐sharing mechanisms.

##### 3.3.3.3 Meaningful Coordination Measurement Requires Patient‐Reported, Holistic and Context‐Sensitive Approaches

Beyond issues of proxies and data quality, respondents identified more fundamental challenges relating to the conceptual validity of several indicators. These concerns centred on ambiguity, context‐dependence, and the risk of misinterpretation.

Several measures were described as lacking clear or consistent meaning. Follow‐up visits could indicate either effective coordination or a breakdown in care; frequent GP attendance might reflect proactive management or unmet need elsewhere; and abnormal test results do not always necessitate action. Similarly, measures such as medication review timing or post‐discharge follow‐up were seen as highly context‐dependent and not universally appropriate. As one participant noted:

Frequency of GP visits might mean ‘waiting well’ but it also could reflect very poor experience.

(R26)

These examples suggest that some indicators do not simply omit important dimensions of coordination but may lack stable interpretability altogether. When used in isolation, they risk misrepresenting care processes unless carefully contextualised within clinical, organisational, and patient‐specific circumstances. This highlights the need for more flexible, condition‐sensitive, and individualised approaches to measurement.

A more fundamental gap identified by respondents was the inability of routine indicators to capture the relational and experiential dimensions of care coordination. Participants consistently emphasised the importance of trust, empathy, communication quality, and how patients feel known, listened to, and supported. Effective coordination was closely linked to person‐centred care practices, including understanding what matters to patients, personalised care planning, involvement of carers and family, and clear, accessible communication.

However, such dimensions cannot be reliably inferred from routine quantitative indicators such as appointment frequency, continuity metrics, referral completion, or medication reviews. As respondents emphasised:

The patient should not be considered a number.

(R22)

It doesn't show if the person feels known, listened to, or supported.

(R25)

These accounts point to a fundamental mismatch between what is easily measurable within routine data systems and what constitutes meaningful, person‐centred coordination. As a result, respondents strongly advocated for the incorporation of direct patient input, particularly through patient‐reported experience measures, to complement routine indicators.

Taken together, these findings indicate that while routine EHR‐derived indicators can provide useful high‐level signals of coordination, they remain constrained by limitations in proxy design, data quality, and conceptual validity. In isolation, they risk offering a partial—and at times misleading—representation of care coordination. Capturing the multidimensional, relational, and person‐centred nature of coordination, therefore, requires a more integrative approach, combining routine data with patient‐reported experience measures and contextualised interpretation.

## 4 Discussion

This study examined whether care coordination for MLTC can be meaningfully measured using routine data. Contributors recognised the value of such measures for monitoring and improvement but emphasised their limitations. Although a feasible set of indicators was identified, these function largely as proxies and do not capture relational aspects of care. Routine data can therefore provide useful system‐level signals but requires cautious interpretation and complementary approaches to reflect what matters to patients and carers.

Our findings reinforce evidence of fragmented care, poor information sharing, and the burden placed on patients and carers to navigate complex systems [3, 4, 5, 7, 18]. For instance, a comprehensive review of 54 studies from 14 countries found that lack of service integration, insufficient coordination across providers, and challenges in delivering person‐centred care were common features of hospital care internationally, underscoring the importance of developing robust approaches to measuring care coordination in MLTC [5]. The present work extends this literature by showing how these challenges are reproduced in measurement. Our preceding scoping review) found substantial heterogeneity across existing coordination measures, with most focusing on organisational processes, healthcare utilisation, continuity within specific settings, or care management activities [15]. In contrast, relatively few measures captured relational, informational, and person‐centred dimensions of coordination, despite these being consistently identified as important by patients and carers. The consultation findings reinforce this mismatch between what is commonly measured and what contributors consider meaningful. Participants placed particular emphasis on relational continuity, accountability, equity, and avoiding burden‐shifting to patients and carers, themes that are often underrepresented in existing measurement frameworks. Patients and carers emphasised being known, supported, and not required to coordinate their own care [45, 46]. These perspectives challenge system‐centric approaches that prioritise efficiency and activity over lived experience. In doing so, the findings align with emerging critiques of coordination metrics that fail to capture the interpersonal, experiential, and longitudinal aspects of care [13], while also providing a structured approach for identifying indicators that better reflect the realities of living with and managing MLTC.

### 4.1 Implications

Routine data–derived measures are scalable and feasible but inherently limited by their reliance on proxy constructs and incomplete capture of lived experience. Their use should therefore be framed as indicative rather than definitive. Advancing measurement will require three conditions: (1) improved data infrastructure, including interoperability and linkage across sectors [47], (2) incorporation of patient‐reported experience data to complement routine indicators and capture relational and person‐centred aspects of coordination [48], and (3) careful attention to interpretation to avoid over‐simplifying complex care processes [49]. Both the scoping review [15] and consultation findings suggest that these aspects are central to the experience of coordinated care but remain poorly represented in routine datasets, which are primarily designed to capture clinical activity and service utilisation [48]. PROMs and PREMs may help address gaps in routine data by capturing communication, information sharing, relational continuity, and patients’ experiences of coordinated care. Evidence suggests these measures are associated with positive health outcomes, although the evidence base is less developed and consistent than that for administrative continuity measures [9, 50]. However, PREMs are influenced by individual expectations and experiences and provide limited insight into the organisational mechanisms underlying coordination. Consequently, patient‐reported measures should be viewed as a complement to, rather than a replacement for, routine indicators within a broader measurement framework; without such an approach, there is a risk that measurement efforts reinforce narrow, system‐centric views of coordination.

Within the emerging MLTC models of care, our findings highlight the need for measurement frameworks that reflect the longitudinal and cross‐sector nature of MLTC care [51]. Incorporating multiple contributor perspectives—particularly the emphasis on relational continuity, accountability, communication, and burden‐shifting—aligns with the principles underpinning integrated care systems [19, 52]. The lower prioritisation of domains of Patient Centredness, such as Self‐management and Community Resources, likely reflects measurement challenges within EHR‐based systems rather than a lack of importance. Addressing this imbalance will require the development of measures that extend beyond healthcare utilisation and better incorporate patient‐reported experiences, along with improved mechanisms for capturing interactions with community and voluntary sector services [53].

### 4.2 Future Research

Future work should focus on the empirical validation of the prioritised measures, including assessments of reliability, construct validity, and sensitivity to change within routine care settings. There is a need to examine how well these indicators capture meaningful variation in coordination and whether they are predictive of outcomes important to patients, such as quality of life, treatment burden, and care experience.

Further methodological development is also required to integrate patient‐reported outcomes and experiences with EHR‐derived measures. Advances in data linkage and digital infrastructure may enable more sophisticated approaches to capturing coordination across sectors, including social care and community services [53]. This is essential to address the identified gap between what is measurable and what matters to patients. Future research should also evaluate how best to combine routine indicators with patient‐reported experience measures and other qualitative approaches. Particular attention is needed to understand the added value, interpretability, and feasibility of multi‐source measurement frameworks, as well as the extent to which they improve the assessment of relational, informational, and person‐centred coordination in MLTC.

Finally, future research should prioritise inclusivity and equity in both measure development and validation. While we engaged a diverse group of contributors, there remains a need to involve groups who are typically underrepresented in research, including those experiencing socioeconomic disadvantage, digital exclusion, or barriers to accessing care. Strengthening co‐production approaches with these populations will be critical to ensuring that future measurement frameworks are both inclusive and generalisable.

### 4.3 Strengths and Limitations

A key strength is its multi‐stage, co‐produced design, integrating perspectives from patients, carers, practitioners, researchers, and data experts. This approach enabled the development of an appraisal framework grounded in real‐world priorities while explicitly incorporating feasibility considerations related to UK data systems. The combination of structured consultations and iterative refinement enhances the robustness and practical relevance of the findings. However, limitations should be acknowledged. Early inclusive mapping required substantial consolidation, and feasibility assessments were constrained by current UK data infrastructure, limiting measurement of relational and experiential aspects. Although engagement was broad, some groups may remain underrepresented, and online methods may have restricted participation. An Equality Impact Assessment was undertaken as part of the wider work of MLTC models of care workstream with the public contributors [54].

A number of professional contributors were based in London. However, this partly reflects the location of national policymaking, commissioning and health system leadership functions, many of which are centred in London‐based government departments, arm's‐length bodies, and national NHS organisations. As our study sought input from individuals involved in the design, implementation and measurement of care coordination at a system level, representation from these organisations was important. Nevertheless, we recognise that priorities, service configurations, and data infrastructures may vary across regions, and broader geographical representation would be valuable in future work to ensure findings reflect the diversity of health and care contexts across the United Kingdom.

Finally, survey responses were anonymous, and participant characteristics were not collected in sufficient detail to enable analyses by consultation group, professional background, contributor role, or lived experience. In addition, the health status question did not distinguish between participants living with a single long‐term condition and those living with MLTC. Consequently, it was not possible to examine whether priorities differed across consultation groups or according to health status. Although this was an intentional design decision, as the survey was intended to support consensus‐building and identify areas of agreement rather than compare perspectives between groups, it limits the extent to which the findings can be interpreted for specific contributor populations. This limitation was partially mitigated through the consultation exercises, which provided richer contextual insights into participants’ views and experiences.

## 5 Conclusion

Measuring care coordination in MLTC using routine data is both necessary and inherently constrained. Contributors support the development of such measures, but only with important caveats regarding validity, data quality, and scope. Routine indicators can provide valuable system‐level signals, but cannot, on their own, capture the full complexity of coordinated care. The central implication is a qualified one: routine data can inform, but not define, coordination. Future work should focus on validating these measures, improving data systems, and integrating patient‐reported perspectives.

## Author Contributions

**Krystal Warmoth:** conceptualisation, writing – original draft, writing – review and editing, formal analysis, funding acquisition. **Nicola Small:** writing – review and editing, formal analysis, project administration, data curation, investigation. **Vanessa Davey:** investigation, writing – review and editing, data curation, formal analysis. **Patrick Burch:** conceptualisation, funding acquisition, supervision. **Alex Thompson:** writing – review and editing. **Jo Butterworth:** methodology, writing – review and editing. **Jonathan Gibb:** writing – review and editing. **Easter Joury:** writing – review and editing. **Steve Callaghan:** writing – review and editing. **Felicity Dewhurst:** funding acquisition, conceptualisation, writing – review and editing, supervision.

## Lived Experience and Public Contribution

Public contributors with lived experience of Multiple Long‐Term Conditions, including carers, were involved throughout all stages of this work, shaping its focus, design, and interpretation. An established Patient and Public Involvement and Engagement panel participated in structured consultations. Public contributors also helped develop the appraisal criteria for assessing measures, ensuring they reflected what matters to patients, including feeling known, supported, and involved in care. They reviewed study materials to improve accessibility and contributed to interpreting findings. Their involvement strengthened the relevance and applicability of the study.

## Conflicts of Interest

The authors declare that they have no known competing financial interests, personal relationships, or other conflicts of interest that could have appeared to influence the work reported in this paper.

## Supporting information


Supporting File 1

Supporting File 2

Supporting File 3

## Data Availability

The data that support the findings of this study are available on request from the corresponding author. The data are not publicly available due to containing information that could compromise the privacy of research participants.
