# Supplementary material for: Can Care Coordination for Multiple Long‐Term Conditions Be Meaningfully Measured Using Routine Data? A Multi‐Stage Consultation
Source: Health Expect. 2026 Oct 3;29(5):e70894. doi: 10.1111/hex.70894 (PMC13633699; doi:10.1111/hex.70894)
Supplement: Supplementary file 1 [file HEX-29-e70894-s001.docx]

ACCORD Follow-up Questions

Start of Block: Default Question Block

**Introduction**

Thank you again for taking part in our survey.

We are developing a way to measure how well care is coordinated for people living with multiple long-term conditions using electronic health records and your input will help shape how care coordination is measured in future research and healthcare improvement work.

In the survey, we identified that a small number of questions did not display correctly for some participants and we would like to gather a small amount of additional information. We would be very grateful if you could also answer these quick 5 rating questions. You only need to answer the rating questions - comments are optional.

This should take around 5-10 minutes to complete.

Thank you for your time.

Very Best Wishes,

ACCORD Research Team

| Page Break |  |
| --- | --- |

**Instructions**

**Care coordination can be broken down into 11 key parts (or activities) and 5 approaches that help us understand and measure how well it works.**

In this short follow-up survey, 3 parts of care coordination and 2 approaches to care coordination will be introduced with an infographic:

1. Accountability - Align Resources with Patient and Population Needs

2. Communication - Interpersonal Communication

3. Communication - Information Transfer

4. Care Management

5. Medication Management

We will then describe one or more ways researchers have tried to measure that part/ approach to care coordination using information recorded in electronic health records.

Sometimes there is more than one possible way to measure that part, and we are interested in your views on whether these approaches make sense. You do not need to understand the technical name of the measures. We are interested in whether the idea behind the measure seems reasonable and meaningful to you.

We will then ask you to:

1. Rate how appropriate you think each measure is for capturing that part of care coordination. 2. Share your thoughts or suggest other ways the part could be measured (this question is optional).

| Page Break |  |
| --- | --- |

**Question 1**
**Accountability - Align resources with patient and population needs**

 

**About this part of coordination:** This is about making sure services and resources are organised fairly and appropriately to meet both individual needs and the needs of the wider population. We have identified two possible measures that could reflect how well resources are aligned with patient and population needs.

Please read both before answering the questions below.

**Option 1: The first measure to consider is the Specialty Intensity Score.**

**• Specialty Intensity Score:** Uses diagnosis information in electronic health record data to estimate how many different specialist physicians a person is likely to need.

**• Why we chose it:** This measure estimates how many different specialists a person may need based on their health conditions. By comparing this expected need with the care people actually receive, it may show whether access to specialist care is fair and appropriate.

**Option 2: The second measure to consider is the Cover Index.**

**• Cover Index:** Uses visit records to measure how much of a person’s time is covered by regular primary care contact, based on the time between visits.

**• Why we chose it**: This measure shows whether someone has regular contact with primary care over time. If there are long gaps between visits, it may suggest that monitoring or follow-up care is missing.

| Page Break |  |
| --- | --- |

**Q1. How appropriate do you think each of these measures is for capturing “Align resources with patient and population needs” using electronic health records?** Please select one option for each row. It is fine if more than one measure seems appropriate.

|  | Very appropriate | Somewhat appropriate | Neither appropriate nor inappropriate | Somewhat inappropriate | Not appropriate | Unsure |
| --- | --- | --- | --- | --- | --- | --- |
| Speciality Intensity Score |  |  |  |  |  |  |
| Cover Index |  |  |  |  |  |  |

| Page Break |  |
| --- | --- |

**Optional Q** If you have any comments about these measures, or suggestions for other ways it could be measured, please share them here. This part of the question is optional.

________________________________________________________________

________________________________________________________________

________________________________________________________________

________________________________________________________________

________________________________________________________________

| Page Break |  |
| --- | --- |

**Question 2** **Communication - Interpersonal Communication**

 
**About this part of coordination:** This is about how clearly healthcare professionals communicate with patients and families and with each other.

**Possible ways to measure this using electronic health records:** We have identified three possible best-fit measures that may reflect interpersonal communication through patterns of continuity in care. Please read all three measure descriptions before answering the questions below.

**Option 1: Continuity of GP Care - Primary Care**

**• What it measures**: Uses visit records to measure the percentage of a person’s GP visits that are with their most frequently seen GP or practice over a set time period (e.g., a year).

**• Why we chose it:** Seeing the same GP regularly may help build understanding and trust between patients and clinicians. This continuity may support clearer communication and better coordination of care.

**Option 2: Specialist Encounter Continuity - Secondary Care**

**• What it measures**: The percentage of a person’s specialist visits that are with their most frequently seen specialist over a set time period.

**• Why we chose it:** If someone sees the same specialist most of the time, it may help that clinician understand their condition and communicate more effectively with other professionals involved in their care.

**Option 3: Known Provider Continuity - Specialist Provider** **• What it measures:** Whether a person sees the same specialist at least twice a year and continues with them over time.

**• Why we chose it:** This measure looks at whether people continue to see the same specialist over time. Long-term relationships with specialists may support clearer communication and more consistent care.

| Page Break |  |
| --- | --- |

**Question 2** **Q2. How appropriate do you think each of these measures is for capturing “Interpersonal Communication” using electronic health records?** Please select one option for each row. It is fine if more than one measure seems appropriate.

|  | Very approriate | Somewhat appropriate | Neither appropriate nor inappropriate | Somewhat inappropriate | Not appropriate | Unsure |
| --- | --- | --- | --- | --- | --- | --- |
| Continuity of GP Care - Primary Care |  |  |  |  |  |  |
| Specialist Encounter Continuity - Secondary Care |  |  |  |  |  |  |
| Known Provider Continuity - Specialist Provider |  |  |  |  |  |  |

| Page Break |  |
| --- | --- |

**Optional Q** If you have any comments about these measures, or suggestions for other ways it could be measured, please share them here. This part of the question is optional.

________________________________________________________________

________________________________________________________________

________________________________________________________________

________________________________________________________________

________________________________________________________________

| Page Break |  |
| --- | --- |

Question 3 **Communication - Information Transfer**

**About this part of coordination:** This activity is about how well healthcare professionals share information with each other to support safe, coordinated care.

Good information transfer helps:

- Ensure important details are not missed
- Reduce duplication or delays
- Identify where coordination is strong and where improvements are needed

This is especially important for people living with multiple long-term conditions, who often move between services and providers.

**Possible ways to measure this using electronic health records:** We have identified two possible measures that may reflect how well information is transferred between professionals. Please read both before answering the question below.

**Option 1: Referral Loop Completeness (Closed-loop Referral)**

**• Referral loop completeness (Closed-loop Referral):** Uses referral and correspondence records to measure the percentage of referrals where a referral is sent, the specialist visit occurs, and feedback is received by the referrer within a set time period.

**• Why we chose it:** This measure shows whether information from a specialist visit is sent back to the doctor who made the referral. This helps ensure everyone involved in a person’s care has the information they need.

**Option 2: Discharge Communication (Timely Discharge Summary)**

**• What it measures:** The percentage of hospital discharges where a summary or transition record is made available to the next care team within a set time period.

**• Why we chose it:** When someone leaves hospital, it is important that their GP or care team receives a summary of what happened. This measure shows whether that information is shared promptly.

| Page Break |  |
| --- | --- |

**Question 3** **Q3. How appropriate do you think each of these measures is for capturing “Information Transfer” using electronic health records?**  Please select one option for each row. It is fine if more than one measure seems appropriate.

|  | Very approriate | Somewhat appropriate | Neither appropriate nor inappropriate | Somewhat inappropriate | Not appropriate | Unsure |
| --- | --- | --- | --- | --- | --- | --- |
| Referral Loop Completeness (Closed-loop Referral) |  |  |  |  |  |  |
| Discharge Communication (Timely Discharge Summary) |  |  |  |  |  |  |

| Page Break |  |
| --- | --- |

**Optional Q** If you have any comments about these measures, or suggestions for other ways it could be measured, please share them here. This part of the question is optional.

________________________________________________________________

________________________________________________________________

________________________________________________________________

________________________________________________________________

________________________________________________________________

| Page Break |  |
| --- | --- |

Question 4 **Coordination Approach: Care Management**

**About this coordination approach:** Care Management is about making sure patients receive the right help from health professionals and support systems.

It includes:

• Ongoing oversight of care

• Coordinating across different services

• Supporting people with complex or multiple long-term conditions

• Using electronic health records and related data, we can assess whether care is being managed in a coordinated and effective way.

**Possible ways to measure this using electronic health records:** We have identified two possible measures that may reflect care management. Please read both before answering the question below.

**Option 1: Fortney Continuity (Case Management Continuity)**

**• What it measures:** Uses routine data to measure whether a person receives ongoing support from the same case manager or care coordinator over a defined period.

**• Why we chose it:** This measure shows whether someone receives ongoing support from the same care coordinator or case manager. Having a consistent person involved may help organise care across different services.

**Option 2: Comorbidity Care and Coordination Indicators**

**• What they measure:** Whether key coordination activities take place for people with multiple long-term conditions — such as follow-up, medication oversight, and communication across providers.

**• Why we chose them:** These indicators show whether important coordination activities are happening for people with multiple long-term conditions, such as follow-up care or communication between services.

| Page Break |  |
| --- | --- |

Question 4 **Q4. How appropriate do you think each of these measures is for capturing “Care Management” using electronic health records?** Please select one option for each row. It is fine if more than one measure seems appropriate.

|  | Very approriate | Somewhat appropriate | Neither appropriate nor inappropriate | Somewhat inappropriate | Not appropriate | Unsure |
| --- | --- | --- | --- | --- | --- | --- |
| Fortney Continuity Measures (Case management Continuity) |  |  |  |  |  |  |
| Comorbidity Care and Coordination Indicators |  |  |  |  |  |  |

| Page Break |  |
| --- | --- |

**Optional Q** If you have any comments about these measures, or suggestions for other ways it could be measured, please share them here. This part of the question is optional.

________________________________________________________________

________________________________________________________________

________________________________________________________________

________________________________________________________________

________________________________________________________________

| Page Break |  |
| --- | --- |

Question 5 **Coordination Approach: Medication Management**

**About this coordination approach:** Medication Management is about keeping people safe when:

• Medicines are changed

• Care moves between settings (for example, from hospital to home)

• People are taking multiple medicines

This is especially important for people living with multiple long-term conditions, who may face higher risks of medication errors, interactions, or treatment burden.

**Possible ways to measure this using electronic health records: We** have identified two possible measures that may reflect medication management. Please read both before answering the question below.

**Option 1: Medication reconciliation within 14 days of discharge/ transition**

• **Medication reconciliation after discharge:** Uses routine data to measure the percentage of care transitions where medications are reviewed and confirmed within 14 days of discharge.

• **Why we chose it:** When medicines change during hospital stays or care transitions, they need to be checked carefully. This measure shows whether medication lists are reviewed and confirmed after discharge.

**Option 2: Polypharmacy review rate**

**• What it measures:** The percentage of people taking multiple medicines who receive a structured medication review within a year.

**• Why we chose it:** People taking many medicines may benefit from regular reviews. This measure shows whether patients with multiple medicines receive structured medication reviews.

| Page Break |  |
| --- | --- |

Question 5 **Q5. How appropriate do you think each of these measures is for capturing “Medication Management” using electronic health records?** Please select one option for each row. It is fine if more than one measure seems appropriate.

|  | Very approriate | Somewhat appropriate | Neither appropriate nor inappropriate | Somewhat inappropriate | Not appropriate | Unsure |
| --- | --- | --- | --- | --- | --- | --- |
| Medication reconciliation within 14 days of discharge/ transition |  |  |  |  |  |  |
| Polypharmacy review rate |  |  |  |  |  |  |

| Page Break |  |
| --- | --- |

**Optional Q** If you have any comments about these measures, or suggestions for other ways it could be measured, please share them here. This part of the question is optional.

________________________________________________________________

________________________________________________________________

________________________________________________________________

________________________________________________________________

________________________________________________________________

| Page Break |  |
| --- | --- |

**Thank you for your time!**

End of survey

End of Block: Default Question Block

Start of Block: Block 1
