# Supplementary material for: Can Care Coordination for Multiple Long‐Term Conditions Be Meaningfully Measured Using Routine Data? A Multi‐Stage Consultation
Source: Health Expect. 2026 Oct 3;29(5):e70894. doi: 10.1111/hex.70894 (PMC13633699; doi:10.1111/hex.70894)
Supplement: Supplementary file 2 [file HEX-29-e70894-s002.docx]

**Supplementary file: Table 1** GRIPP2 short form

| Section and topic | Item | Reported on page No |
| --- | --- | --- |
| 1: Aim | Report the aim of PPIE in the study | 4,6-7 |
| 2: Methods | Provide a clear description of the methods used for PPI in the study | 6-10, Tables 1-2 |
| 3: Study results | Outcomes—Report the results of PPIE in the study, including both positive and negative outcomes | 11-21, Tables 3-4 |
| 4: Discussion and conclusions | Outcomes—Comment on the extent to which PPIE influenced the study overall. Describe positive and negative effects | 21-23 |
| 5: Reflections/critical perspective | Comment critically on the study, reflecting on the things that went well and those that did not, so others can learn from this experience | 23-24 |

Note: PPIE=patient and public involvement and engagement
