# Supplementary material for: Can Care Coordination for Multiple Long‐Term Conditions Be Meaningfully Measured Using Routine Data? A Multi‐Stage Consultation
Source: Health Expect. 2026 Oct 3;29(5):e70894. doi: 10.1111/hex.70894 (PMC13633699; doi:10.1111/hex.70894)
Supplement: Supplementary file 3 [file HEX-29-e70894-s003.docx]

ACCORD-MLTC Stakeholder Survey

Start of Block: Section 1: Introduction and consent

Section 1 **Introduction**

**We are developing a way to measure how well care is coordinated for people living with multiple long-term conditions using electronic health records.**

This survey is part of our Patient and Public Involvement and Engagement and stakeholder work. Your views will help us make sure this research reflects what matters most to patients and the public. 

There are no right or wrong answers - we are interested in your perspective on the different parts to measure care coordination and our approach to it. 

The survey takes about 20 minutes if you simply select responses. It may take a little longer (around 30-60 minutes), if you choose to add comments in the text boxes (these are optional). Most questions simply involve selecting a response.

You will receive £30 Love2Shop e-voucher as a thank you for your time.

• Participation is voluntary
• Responses are anonymous
• Data will only be used for research and reporting

Your input will help shape how care coordination is measured in future research and healthcare improvement work.

Thank you very much.

| Page Break |  |
| --- | --- |

Section 1: **Q1. Do you agree to take part in this survey?**

- Yes, I agree
- No, I do not agree

Skip To: End of Survey If Consent = No, I do not agree

End of Block: Section 1: Introduction and consent

Start of Block: Section 2: Feedback on each atlas activity

Section 2 **Care coordination can be broken down into 12 key parts (or activities) that help us understand and measure how well it works.**

Each part will be introduced with an infographic. We will then describe one or more ways researchers have tried to measure that part using information recorded in electronic health records.

Sometimes there is more than one possible way to measure a part, and we are interested in your views on whether these approaches make sense. You do not need to understand the technical name of the measures. We are interested in whether the idea behind the measure seems reasonable and meaningful to you.

We will then ask you to:
1. Rate how appropriate you think each measure is for capturing that part of care coordination.
2. Share your thoughts or suggest other ways the part could be measured (optional).

| Page Break |  |
| --- | --- |

Section 2 **First part of coordination: 1a. Accountability - Establish Accountability or Negotiate Responsibility**

**About this part of coordination:** Establishing Accountability or Negotiating Responsibility is about making sure there is clarity on which healthcare professional or service is responsible for coordinating a person’s care. People should know who to turn to if they have questions or problems.

**Best fit measure using electronic health records:** **Usual Provider of Care**

- **Usual Provider of Care:** Uses visit records to measure the percentage of a person’s visits that are with the same clinician or practice over a set time period (e.g., a year).
- **Why we chose it:** This measure shows whether a person usually sees the same GP or GP practice. If most visits are with the same provider, it suggests there is clear responsibility for coordinating their care. If visits are spread across many providers, this may indicate less clarity about who is overseeing care.

Section 2 **Q2. Do you think this is an appropriate way of measuring “Establish Accountability or Negotiate Responsibility” using electronic health records?**

- Yes
- No
- Unsure

Section 2 **Q3. If you have any comments about this measure, or suggestions for other ways it could be measured, please share them here (optional).**

________________________________________________________________

________________________________________________________________

________________________________________________________________

________________________________________________________________

________________________________________________________________

| Page Break |  |
| --- | --- |

Section 2 **1b. Accountability - Align resources with patient and population needs**

**About this part of coordination:** This is about making sure services and resources are organised fairly and appropriately to meet both individual needs and the needs of the wider population.

We have identified two possible measures that could reflect how well resources are aligned with patient and population needs. Please read both before answering the questions below.

**Option 1: The first measure to consider is the Specialty Intensity Score.**

- **Specialty Intensity Score:** Uses diagnosis information in electronic health record data to estimate how many different specialist physicians a person is likely to need.
- **Why we chose it:** This measure estimates how many different specialists a person may need based on their health conditions. By comparing this expected need with the care people actually receive, it may show whether access to specialist care is fair and appropriate.
   
  **Option 2: The second measure to consider is the Cover Index.**
- **Cover Index:** Uses visit records to measure how much of a person’s time is covered by regular primary care contact, based on the time between visits.
- **Why we chose it:** This measure shows whether someone has regular contact with primary care over time. If there are long gaps between visits, it may suggest that monitoring or follow-up care is missing.

Section 2: **Q4. How appropriate do you think each of these measures is for capturing “Align resources with patient and population needs” using electronic health records?**

**Please rate each measure separately. It is fine if more than one measure seems appropriate.**

|  | Very appropriate | Somewhat appropriate | Neither appropriate nor inappropriate | Somewhat inappropriate | Not appropriate | Unsure |
| --- | --- | --- | --- | --- | --- | --- |
| Speciality Intensity Score |  |  |  |  |  |  |
| Cover Index |  |  |  |  |  |  |

Section 2 **Q5. If you have any comments about these measures, or suggestions for other ways it could be measured, please share them here (optional).**

________________________________________________________________

________________________________________________________________

________________________________________________________________

________________________________________________________________

________________________________________________________________

| Page Break |  |
| --- | --- |

Section 2 **Second part of coordination: 2a. Communication - Interpersonal Communication**

**About this part of coordination:** This is about how clearly healthcare professionals communicate with patients and families and with each other.

**Possible ways to measure this using electronic health records:** We have identified three possible best-fit measures that may reflect interpersonal communication through patterns of continuity in care. Please read all three measure descriptions before answering the questions below.

**Option 1: Continuity of GP Care - Primary Care**

- **What it measures:** Uses visit records to measure the percentage of a person’s GP visits that are with their most frequently seen GP or practice over a set time period (e.g., a year).
- **Why we chose it:** Seeing the same GP regularly may help build understanding and trust between patients and clinicians. This continuity may support clearer communication and better coordination of care.
   
  **Option 2: Specialist Encounter Continuity - Secondary Care**
- **What it measures:** The percentage of a person’s specialist visits that are with their most frequently seen specialist over a set time period.
- **Why we chose it:** If someone sees the same specialist most of the time, it may help that clinician understand their condition and communicate more effectively with other professionals involved in their care.
   
  **Option 3: Known Provider Continuity - Specialist Provider**
- **What it measures:** Whether a person sees the same specialist at least twice a year and continues with them over time.
- **Why we chose it:** This measure looks at whether people continue to see the same specialist over time. Long-term relationships with specialists may support clearer communication and more consistent care.

Section 2 **Q6. How appropriate do you think each of these measures is for capturing “Interpersonal Communication” using electronic health records?**

**Please rate each measure separately. It is fine if more than one measure seems appropriate.**

|  | Very appropriate | Somewhat appropriate | Neither appropriate nor inappropriate | Somewhat inappropriate | Not appropriate | Unsure |
| --- | --- | --- | --- | --- | --- | --- |
| Continuity of GP Care - Primary Care |  |  |  |  |  |  |
| Specialist Encounter Continuity - Secondary Care |  |  |  |  |  |  |
| Known Provider Continuity - Specialist Provider |  |  |  |  |  |  |

Section 2 **Q7 If you have any comments about these measures, or suggestions for other ways it could be measured, please share them here (optional).**

________________________________________________________________

________________________________________________________________

________________________________________________________________

________________________________________________________________

________________________________________________________________

| Page Break |  |
| --- | --- |

Section 2 **2b. Communication - Information Transfer**

 

**About this part of coordination:** This activity is about how well healthcare professionals share information with each other to support safe, coordinated care.

Good information transfer helps:

- Ensure important details are not missed
- Reduce duplication or delays
- Identify where coordination is strong and where improvements are needed

This is especially important for people living with multiple long-term conditions, who often move between services and providers.

**Possible ways to measure this using electronic health records:** We have identified two possible measures that may reflect how well information is transferred between professionals. Please read both before answering the question below.

**Option 1: Referral Loop Completeness (Closed-loop Referral)**

- **Referral loop completeness (Closed-loop Referral):** Uses referral and correspondence records to measure the percentage of referrals where a referral is sent, the specialist visit occurs, and feedback is received by the referrer within a set time period.
- **Why we chose it:** This measure shows whether information from a specialist visit is sent back to the doctor who made the referral. This helps ensure everyone involved in a person’s care has the information they need.

**Option 2: Discharge Communication (Timely Discharge Summary)**

- **What it measures:** The percentage of hospital discharges where a summary or transition record is made available to the next care team within a set time period.
- **Why we chose it:** When someone leaves hospital, it is important that their GP or care team receives a summary of what happened. This measure shows whether that information is shared promptly.

Section 2 **Q8. How appropriate do you think each of these measures is for capturing "Information Transfer” using electronic health records?**

Please rate each measure separately. It is fine if more than one measure seems appropriate.

|  | Very appropriate | Somewhat appropriate | Neither appropriate nor inappropriate | Somewhat inappropriate | Not appropriate | Unsure |
| --- | --- | --- | --- | --- | --- | --- |
| Referral loop completeness (closed-loop referral) |  |  |  |  |  |  |
| Discharge communication |  |  |  |  |  |  |

Section 2 **Q9. If you have any comments about these measures, or suggestions for other ways it could be measured, please share them here (optional).**

________________________________________________________________

________________________________________________________________

________________________________________________________________

________________________________________________________________

________________________________________________________________

| Page Break |  |
| --- | --- |

Section 2 **Third part of coordination: 3a. Transitions of care: Across settings**

**About this part of coordination:** This is about making sure care is coordinated when people move between services, settings, or levels of care. For example, from hospital back to home, or from specialist care back to the GP.

**Best fit measure using electronic health records: Follow-Up After Hospitalisation**

- **Follow-up after hospitalisation:** Uses routine data to measure the percentage of hospital discharges followed by a primary care, outpatient, or mental health visit within a set time period (e.g., 7 or 30 days).
- **Why we chose it:** After leaving hospital, timely follow-up with a GP or specialist can help prevent problems or complications. This measure shows whether that follow-up happens.

Section 2 **Q10 Do you think this is an appropriate way of measuring “Across settings” using electronic health records?**

- Yes
- No
- Unsure

Section 2 **Q11 If you have any comments about this measure, or suggestions for other ways it could be measured, please share them here (optional).**

________________________________________________________________

________________________________________________________________

________________________________________________________________

________________________________________________________________

________________________________________________________________

| Page Break |  |
| --- | --- |

Section 2 **Transitions of care: As coordination needs change**

**About this part of coordination:** This is about making sure care is coordinated when people move between services, settings, or levels of care. For example, when their needs change (e.g., new diagnosis).

**Best fit measure using electronic health records: Cover Index**

- **Cover Index:** Uses visit records to measure how much of a person’s time is ‘covered’ by regular primary care contact, based on the time between visits.
- **Why we chose it:** Regular contact with healthcare professionals can help detect changes in a person’s health. This measure highlights when there are long gaps between visits that might mean changes in health are missed.

Section 2 **Q12 Do you think this is an appropriate way of measuring “As coordination needs change” using electronic health records?**

- Yes
- No
- Unsure

Section 2 **Q13 If you have any comments about this measure, or suggestions for other ways it could be measured, please share them here (optional).**

________________________________________________________________

________________________________________________________________

________________________________________________________________

________________________________________________________________

________________________________________________________________

| Page Break |  |
| --- | --- |

Section 2 **Fourth part of coordination: 4a. Care planning: Assess needs and goals**  
  

**About this part of coordination:** This is about identifying what matters most to a person—their health needs, preferences, and personal goals. Care should be shaped around these needs and goals.

**Best fit measure using electronic health records: Specialty Intensity Score**

- **Specialty Intensity Score:** Uses diagnosis information in routine data to estimate how many different specialist clinicians a person is likely to need.
- **Why we chose it:** This measure estimates how many specialists a person may need based on their health conditions. It can help show whether people with complex needs are receiving the level of care they require.

Section 2 **Q14 Do you think this is an appropriate way of measuring “Assess needs and goals” using electronic health records?**

- Yes
- No
- Unsure

Section 2 **Q15 If you have any comments about this measure, or suggestions for other ways it could be measured, please share them here (optional).**

________________________________________________________________

________________________________________________________________

________________________________________________________________

________________________________________________________________

________________________________________________________________

| Page Break |  |
| --- | --- |

Section 2 **4b. Care planning: Monitor, follow up, and respond to change**

**About this part of coordination:** this is about regularly reviewing a person’s health and adapting care as needed—for example, following up on test results, checking whether treatments are working, and adjusting the plan when things change.

**Best fit measure using electronic health records: Abnormal results follow-up**

- **Abnormal results follow-up:** Uses test and clinical records to measure the percentage of abnormal results that receive timely follow-up action, such as clinician review, patient contact, repeat testing, referral, or treatment change.
- **Why we chose it:** When a test result is abnormal, it is important that action is taken. This measure shows whether abnormal results are followed up in a timely way.

Section 2 **Q16 Do you think this is an appropriate way of measuring “Monitor, follow up, and respond to change” using electronic health records?**

- Yes
- No
- Unsure

Section 2 **Q17 If you have any comments about this measure, or suggestions for other ways it could be measured, please share them here (optional).**

________________________________________________________________

________________________________________________________________

________________________________________________________________

________________________________________________________________

________________________________________________________________

| Page Break |  |
| --- | --- |

Section 2 **Fifth part of coordination: 5a. Patient Centredness - Support Self-Management Goals**

**About this part of coordination:** This activity is about helping people manage their health day-to-day - for example, supporting them with medication, lifestyle changes, or access to self-help resources.

In our review, we did not find any best-fit measures using electronic health records that capture this kind of support. This means there is an important gap.

Section 2 **Q18 What information in electronic health records could show that people are being supported to manage their own health and care?**

For example, this might include whether:

- people receive education, coaching, or training
- support is tailored to their needs
- carers or family are involved where appropriate
- people are helped to navigate services

Please share any ideas or examples in the box below.

________________________________________________________________

________________________________________________________________

________________________________________________________________

________________________________________________________________

________________________________________________________________

| Page Break |  |
| --- | --- |

Section 2 **5b. Patient Centredness - Link to Community Resources**

**About this part of coordination:** This activity is about connecting people to support outside healthcare, such as social care, charities, community groups, or practical help in daily life.

This might include support with:

- Benefits or financial advice
- Housing or practical support
- Social activities or peer support
- Education or employment
- Transport, food, or wellbeing

In our review, we did not find any best-fit measures that show whether people are linked to these kinds of services. This is an important gap.

**Q19. What information in electronic health records could show that people are being linked to community or non-medical support?**

For example, you may want to think about:

- Whether people are given information about local services
- Whether they are referred or actively connected to these services
- Whether support matches what matters most to the person

Please tell us your suggestions for a measure to capture patient-centeredness in electronic health records in the text box below.

________________________________________________________________

________________________________________________________________

________________________________________________________________

________________________________________________________________

________________________________________________________________

End of Block: Section 2: Feedback on each atlas activity

Start of Block: Section 3: Prioritising the activities

Section 3: **Q20. Now that you’ve reviewed these parts of coordination, we’d like to know which you think are most important to measure.** Please rank the parts of coordination in order of importance using the arrows [shown on the screen] to assign numbers, where 1 = most important.

1. ______ Accountability: Establish Accountability or Negotiate Responsibility
2. ______ Accountability: Align Resources with Patient Population Needs
3. ______ Communication: Interpersonal Communication
4. ______ Communication: Information Transfer
5. ______ Transitions of Care: Across Settings
6. ______ Transitions of Care: As Care Needs Arise
7. ______ Care Planning: Assess Needs and Goals
8. ______ Care Planning: Create a Proactive Plan of Care
9. ______ Care Planning: Monitor, Follow-up, and Respond to Change
10. ______ Patient-Centredness: Support Self-Management Goals
11. ______ Patient Centredness: Link to Community Resources

End of Block

Start of Block: Section 4: Feedback on each atlas approach

Section 4  **Care coordination can also be divided up into a number of different approaches.**

In this section, we are going to describe each of these approaches and show one or more ways researchers have tried to measure it using electronic health records.

You will then be asked:

1. How appropriate you think each measure is for capturing this approach to care coordination.
2. To share your thoughts or suggest other ways it could be measured (optional).

Most questions simply ask you to select a response, and most of the comment boxes are optional.

You don’t need to understand the technical name of the measure. We are interested in whether the idea makes sense to you.

| Page Break |  |
| --- | --- |

Section 4 **Coordination Approach 1. Teamwork - Interdisciplinary collaboration**

 

**About this coordination approach:** This approach is about how well different professionals collaborate to care for the same patient.

**Best fit measure using electronic health records: Care Density**

- **Care Density:** Uses routine data to measure how connected a person’s care providers are, based on how often they share patients. If doctors see many of the same patients, they’re more likely to talk and coordinate.
- **Why we chose it:** If clinicians often care for the same patients, they may be more likely to communicate and coordinate their work. This measure looks at how connected a person’s care providers are.

Section 4 **Q21 Do you think this is an appropriate way of measuring “Teamwork - Interdisciplinary collaboration” using electronic health records?**

- Yes
- No
- Unsure

Section 4 **Q22 If you have any comments about this measure, or suggestions for other ways it could be measured, please share them here (optional).**

________________________________________________________________

________________________________________________________________

________________________________________________________________

________________________________________________________________

________________________________________________________________

| Page Break |  |
| --- | --- |

Section 4 **Coordination Approach 2. Care Management**

**About this coordination approach:** Care Management is about making sure patients receive the right help from health professionals and support systems.

It includes:

- Ongoing oversight of care
- Coordinating across different services
- Supporting people with complex or multiple long-term conditions
- Using electronic health records and related data, we can assess whether care is being managed in a coordinated and effective way.

**Possible ways to measure this using electronic health records:** We have identified two possible measures that may reflect care management. Please read both before answering the question below.

**Option 1: Fortney Continuity (Case Management Continuity)**

- **What it measures:** Uses routine data to measure whether a person receives ongoing support from the same case manager or care coordinator over a defined period.
- **Why we chose it:** This measure shows whether someone receives ongoing support from the same care coordinator or case manager. Having a consistent person involved may help organise care across different services.
   
  **Option 2: Comorbidity Care and Coordination Indicators**
- **What they measure:** Whether key coordination activities take place for people with multiple long-term conditions — such as follow-up, medication oversight, and communication across providers.
- **Why we chose them:** These indicators show whether important coordination activities are happening for people with multiple long-term conditions, such as follow-up care or communication between services.

Section 3 **Q23. How appropriate do you think each of these measures is for capturing “Care Management” using electronic health records?**

**Please rate each measure separately. It is fine if more than one measure seems appropriate.**

|  | Very appropriate | Somewhat appropriate | Neither appropriate nor inappropriate | Somewhat inappropriate | Not appropriate | Unsure |
| --- | --- | --- | --- | --- | --- | --- |
| Fortney Continuity Measures (Case Management Continuity) |  |  |  |  |  |  |
| Comorbidity Care and Coordination Indicators |  |  |  |  |  |  |

Section 4 **Q24. Please rate each measure separately. It is fine if more than one measure seems appropriate.**

________________________________________________________________

________________________________________________________________

________________________________________________________________

________________________________________________________________

________________________________________________________________

| Page Break |  |
| --- | --- |

Section 4 **Coordination Approach 3. Medication Management**

**About this coordination approach:** Medication Management is about keeping people safe when:

- Medicines are changed
- Care moves between settings (for example, from hospital to home)
- People are taking multiple medicines

This is especially important for people living with multiple long-term conditions, who may face higher risks of medication errors, interactions, or treatment burden.

**Possible ways to measure this using electronic health records:** We have identified two possible measures that may reflect medication management. Please read both before answering the question below.

**Option 1: Medication reconciliation within 14 days of discharge/ transition**

- **Medication reconciliation after discharge:** Uses routine data to measure the percentage of care transitions where medications are reviewed and confirmed within 14 days of discharge.
- **Why we chose it**: When medicines change during hospital stays or care transitions, they need to be checked carefully. This measure shows whether medication lists are reviewed and confirmed after discharge.

**Option 2: Polypharmacy review rate**

- **What it measures:** The percentage of people taking multiple medicines who receive a structured medication review within a year.
- **Why we chose it:** People taking many medicines may benefit from regular reviews. This measure shows whether patients with multiple medicines receive structured medication reviews.

Section 4 **Q25. How appropriate do you think each of these measures is for capturing “Medication Management” using electronic health records?**

Please rate each measure separately. It is fine if more than one measure seems appropriate.

|  | Very appropriate | Somewhat appropriate | Neither appropriate nor inappropriate | Somewhat inappropriate | Not appropriate | Unsure |
| --- | --- | --- | --- | --- | --- | --- |
| Medication reconciliation within 14 days of discharge/ transition |  |  |  |  |  |  |
| Polypharmacy review rate |  |  |  |  |  |  |

Section 4 **Q26. If you have any comments about these measures, or suggestions for other ways it could be measured, please share them here (optional).**

________________________________________________________________

________________________________________________________________

________________________________________________________________

________________________________________________________________

________________________________________________________________

| Page Break |  |
| --- | --- |

Section 4 **Coordination Approach 4. Health IT-enabled coordination**

**About this coordination approach:** This approach is about how information technology tools such as electronic health records, health information exchanges, and other digital systems support coordination by enabling information sharing and continuity across settings.

**Best fit measure using electronic health records: Health Information Exchange / Electronic Medical Record Use**

- **Electronic Health Information Exchange / Electronic Medical Record use:** Uses system records to measure how often clinicians look up patient information from other organisations and share information electronically.
- **Why we chose it:** Electronic systems can help clinicians access and share patient information across services. This measure shows how often these systems are used to support coordination.

Section 4 **Q27 Do you think this is an appropriate way of measuring “Health IT-enabled coordination” using electronic health records?**

- Yes
- No
- Unsure

Section 4 **Q28 If you have any comments about this measure, or suggestions for other ways it could be measured, please share them here (optional).**

________________________________________________________________

________________________________________________________________

________________________________________________________________

________________________________________________________________

________________________________________________________________

| Page Break |  |
| --- | --- |

Section 4 **Coordination Approach 5. Health Care Home**

**About this coordination approach:** This approach is about whether patients have a consistent, central source of care that coordinates all aspects of their health management.

**Best fit measure using electronic health records: Medical group continuity (attribution stability)**

- **Medical group continuity (attribution stability):** Uses routine data to measure how consistently a person is linked to the same GP practice or care organisation over time.
- **Why we chose it:** This measure shows whether a person is consistently linked to the same GP practice or care organisation. Having a stable place responsible for care may support better coordination.

Section 4 **Q29 Do you think this is an appropriate way of measuring “Health Care Home” using electronic health records?**

- Yes
- No
- Unsure

Section 4 **Q30 If you have any comments about this measure, or suggestions for other ways it could be measured, please share them here (optional).**

________________________________________________________________

________________________________________________________________

________________________________________________________________

________________________________________________________________

________________________________________________________________

| Page Break |  |
| --- | --- |

Section 4 **Relational Continuity**
 


**About Relational Continuity:** refers to an ongoing, trusting relationship between a person and their care provider over time, so the provider knows them well and takes responsibility for their care.

**Best fit measure using electronic health records: Known Provider Continuity – personal provider**

- **Known Provider Continuity – personal provider:** this measure checks whether patients continue to see a “known” doctor from year to year, reflecting relationship stability.
- **Why we chose it:** seeing the same doctor over time may help build trust and understanding. This measure looks at whether patients continue to see the same clinician from year to year.

Section 4 **Q31. Do you think this is an appropriate way of measuring “Relational Continuity” using electronic health records?**

- Yes
- No
- Unsure

Section 4 **Q32. If you have any comments about this measure, or suggestions for other ways it could be measured, please share them here (optional).**

________________________________________________________________

________________________________________________________________

________________________________________________________________

________________________________________________________________

________________________________________________________________

| Page Break |  |
| --- | --- |

Section 4 **Q33. Now that you’ve reviewed these approaches, we’d like to know which you think are most important to measure.** Please rank the approaches to coordination in order of importance using the arrows [shown on the screen] to assign numbers, where 1 = most important.

1. ______ Teamwork (Interdisciplinary collaboration)
2. ______ Care Management
3. ______ Medication Management
4. ______ Health IT-enabled coordination
5. ______ Health Care Home

End of Block

Start of Block: Section 5: Final reflections

Section 5 **Q34. Are there any other important aspects of care coordination that we should measure?**

________________________________________________________________

________________________________________________________________

________________________________________________________________

________________________________________________________________

________________________________________________________________

Section 5 **Q35. Do you have suggestions about how electronic health records could better capture people’s experiences of care coordination?**

________________________________________________________________

________________________________________________________________

________________________________________________________________

________________________________________________________________

________________________________________________________________

End of Block: Section 5: Final reflections

Start of Block: Questions about you (optional)

Section 6: The following questions are **optional.** They help us understand who took part in this survey and whether we heard from a diverse range of people.

| Page Break |  |
| --- | --- |

Section 6 **Q1. What is your age?** (Years) Please choose on response from the multiple choice age bands.

- 18-24
- 25-34
- 35-44
- 45-54
- 55-64
- 65-74
- 75+
- Prefer not to say

| Page Break |  |
| --- | --- |

Section 6 **Q2. What is your gender?**

- Female
- Male
- Non-binary / third gender
- Prefer to self-describe
- Prefer not to say

| Page Break |  |
| --- | --- |

Section 6 **Q3. Do you have one or more long-term health conditions?**

- Yes
- No
- Prefer not to say

| Page Break |  |
| --- | --- |

Section 6 **Q4. Which of the following best describes your ethnic group?**

- English, Welsh, Scottish, Northern Irish or British
- Irish
- Gypsy or Irish Traveller
- Roma
- Any other White background
- White and Black Caribbean
- White and Black African
- White and Asian
- Any other Mixed or Multiple ethnic background
- Indian
- Pakistani
- Bangladeshi
- Chinese
- Any other Asian background
- African
- Caribbean
- Any other Black, African or Caribbean background
- Arab
- Any other ethnic group
- Prefer not to say

End of Block: Questions about you (optional)

**Thank you for sharing your views!** 

Your feedback will help us to create a better measure of care coordination for people with multiple long-term conditions.

End of Survey.
